# Controlling Circularly Polarized Luminescence Using Helically Structured Chiral Silica as a Nanosized Fused Quartz Cell

**DOI:** 10.1021/jacsau.3c00390

**Published:** 2023-09-15

**Authors:** Hinari Sakai, Tsz-Ming Yung, Tomoki Mure, Naoki Kurono, Syuji Fujii, Yoshinobu Nakamura, Teruaki Hayakawa, Ming-Chia Li, Tomoyasu Hirai

**Affiliations:** † Department of Applied Chemistry, Faculty of Engineering and Graduate School of Engineering, Osaka Institute of Technology, 5-16-1 Omiya, Asahi-ku, Osaka 535-8585, Japan; ‡ Department of Biological Science and Technology, Center for Intelligent Drug Systems and Smart Bio-devices (IDS2B), National Yang Ming Chiao Tung University, Hsinchu 30010, Taiwan; § Department of Materials Science and Engineering, School of Materials and Chemical Technology, Tokyo Institute of Technology, 2-12-1-S8-36 Ookayama, Meguro-ku, Tokyo 152-8552, Japan

**Keywords:** polyhedral oligomeric silsesquioxane, stereoregularity, silica, circularly polarized luminescence, helical structure, quartz cell

## Abstract

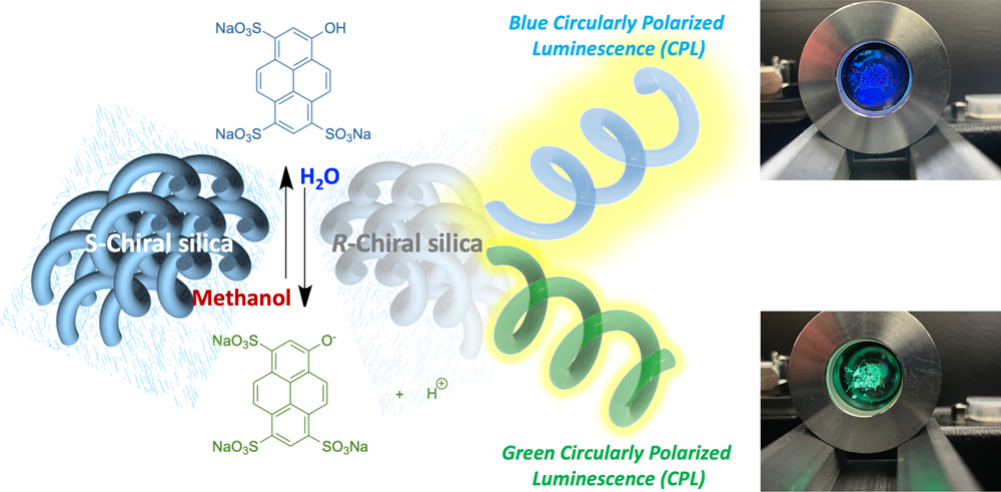

Circularly polarized luminescence (CPL) is typically achieved with a chiral luminophore. However, using a helical nanosized fused quartz cell consisting of chiral silica, we could control the wavelength and helical sense of the CPL of an achiral luminophore. Chiral silica with a helical nanostructure was prepared by calcining a mixture of polyhedral oligomeric silsesquioxane (POSS)-functionalized isotactic poly(methacrylate) (*it*-PMAPOSS) and a small amount of chiral dopant. The chiral silica encapsulated functional molecules, including luminophores, along the helical nanocavity, leading to induced circular dichroism (ICD) and induced circularly polarized luminescence (iCPL). Because chiral silica can act as a helical nanosized fused quartz cell, it can encapsulate not only the luminophore but also solvent molecules. By changing the solvent in the luminophore-containing nanosized fused quartz cell, the wavelength of the CPL was controlled. This method provides an effective strategy for designing novel CPL-active materials.

Silica-based chiral materials have attracted considerable attention in various fields such as catalysis, templating, and chiral recognition. Chiral silica precursors are typically prepared via a sol–gel reaction using tetraethoxysilane with a chiral surfactant^1−3^ or a mixture of polyethylenimine and tartaric acid.^4^ Chiral silica prepared from a chiral surfactant forms a well-ordered helical structure. Although chiral silica prepared from polyethylenimine/tartaric acid does not form a specific helical structure, it does exhibit chiral optical properties.^5^ Because silica-based materials do not absorb light in the UV and visible regions, the helical nanocavities in chiral silica are expected to act as “nano quartz cells” for the circularly polarized luminescence (CPL) of encapsulated solvent and luminophore molecules. Photoluminescence (PL) and CPL are strongly affected by the solvent used. Consequently, if chiral silica can encapsulate both achiral luminophores and solvents within the helical nanocavity, the wavelength of the CPL could be controlled, and self-aggregation-caused quenching (ACQ) could be prevented. Although chiral silica has been used as a host for CPL , accommodating both solvent and luminophore molecules within the chiral silica has not yet been achieved. Helically structured chiral silica typically ranges in length from a few micrometers to ∼100 nm, with helical pores 2–3 nm in diameter.^3,6^ However, this size may be unsuitable for accommodating solvent molecules due to the lack of a nanoscale confinement effect.

Polyhedral oligomeric silsesquioxanes (POSS) are organic–inorganic hybrid materials that convert into silica when exposed to O_2_ plasma or calcined at high temperatures.^7−10^ Various ordered bulk and thin-film nanostructures have been prepared using POSS-containing block copolymers,^11−14^ giant molecules,^15^ and supramolecular compounds.^16^ POSS-containing block copolymers are used to obtain conventional lamellar, cylindrical, and spherical nanostructures, whereas giant POSS-containing molecules are used to prepare gyroid and Frank–Kasper phases. Although natural polymers, including DNA and RNA, form helical structures, the arrangement of POSS in a helical configuration remains challenging. We first reported the control of the preferred-handed helical conformation using an isotactic POSS-functionalized methacrylate polymer (*it*-PMAPOSS) in the presence of small amounts of chiral dopants. The helical structure was maintained during the calcination process, resulting in chiral silica with a helical structure comprising subnanometer-diameter structures.^17^ The small helical nanocavity of the *it*-PMAPOSS-based chiral silica may act as a nanosized fused quartz cell and enable the encapsulation of both luminophore and solvent molecules. Herein we report the control of the CPL wavelength using chiral silica to encapsulate an achiral luminophore and solvent molecules in a helical nanosized quartz cell.

Figure 1a shows the chemical structure of *it*-PMAPOSS. The number-average molecular weight (*M*_n_) and polydispersity index (*Đ*) were 8000 and 1.24, respectively. The stereoregularity of *it*-PMAPOSS was evaluated by using ^13^C NMR spectroscopy. The signals at ∼45 ppm were assigned to meso–meso (*mm*), meso–racemo (*mr*), and racemo–racemo (*rr*) couplings from low to high magnetic field values, respectively. The ratio of *mm*, *mr*, and *rr* in *it*-PMAPOSS was 97:2:1. To control the helical structure, *it*-PMAPOSS and (*S*)-(−)- or (*R*)-(+)-5,5′,6,6′,7,7′,8,8′-octahydro-1,1′-bi-2-naphthol (BN) were mixed at a MAPOSS/BN molar ratio of 0.1 in toluene. The solution was annealed at 90 °C for 2 h, and the toluene was subsequently slowly evaporated. The solid sample was calcined at 620 °C, resulting in chiral silica.^17^Figure 1b,c show the transmission electron microscopy (TEM) image and vibrational circular dichroism (VCD) spectra, respectively, of the chiral silica prepared from *it*-PMAPOSS with enantiomeric BNs. The helical structure was evident from the TEM image. In the VCD spectrum, a clear split-type Cotton effect was observed for the peak between 1000 and 1240 cm^–1^, which was assigned to the Si–O–Si stretching vibration. The Cotton effect between the *it*-PMAPOSS/enantiomeric BN chiral silicas exhibited a mirror relationship. Moreover, no other organic peaks were observed, indicating that chiral silica with a helical structure was obtained.^17^ The chiral silicas prepared from *it*-PMAPOSS/(*S*)-BN and *it*-PMAPOSS/(*R*)-BN are denoted as *S*-chiral and *R*-chiral silica, respectively.

**Figure 1:**
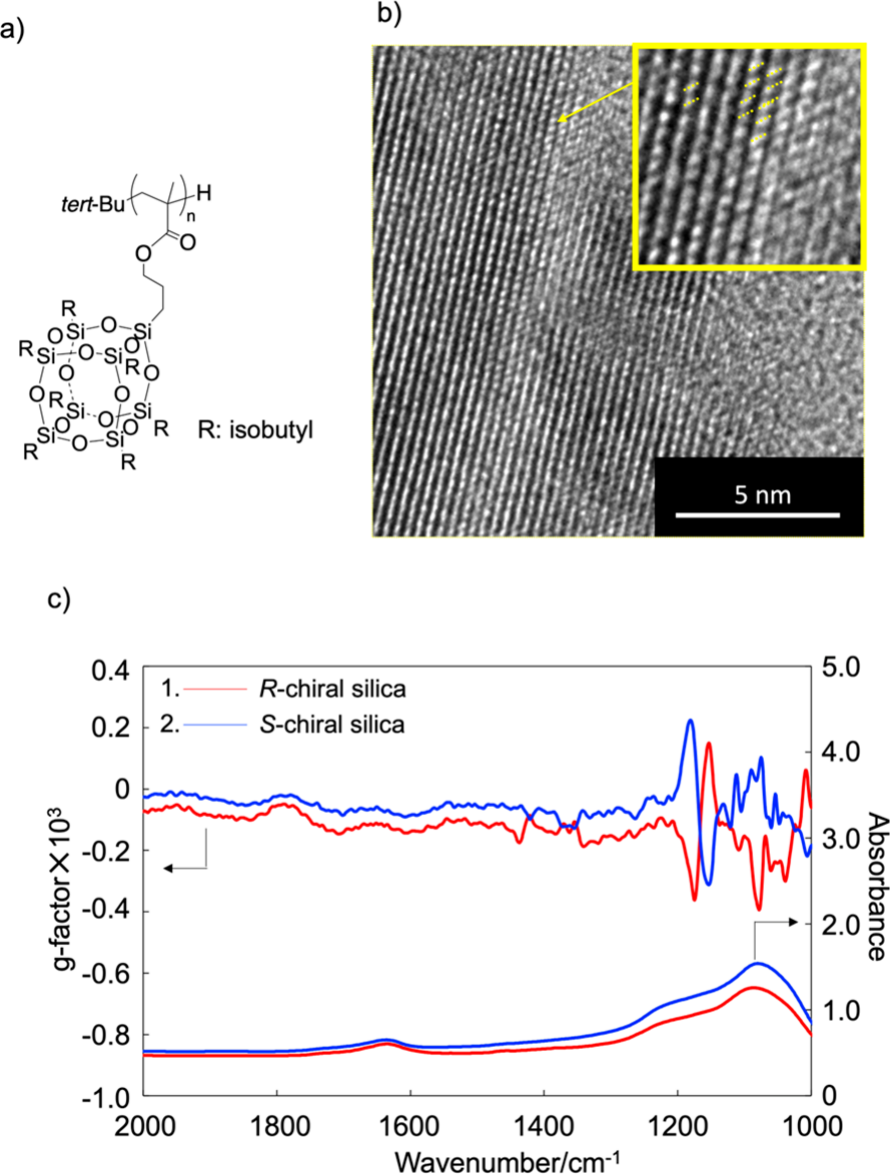
(a) Chemical structure of *it*-PMAPOSS. (b) TEM image of chiral silica prepared from (*R*)-BN. The inset shows a highly magnified image. (c) VCD spectra of chiral silica prepared from *it*-PMAPOSS with enantiomeric BN.

To evaluate the encapsulation behavior of the helical nanocavity in chiral silica, phenol was introduced. Chiral silica was immersed in a phenol/hexane solution for 14 h and then collected by centrifugation. The resulting chiral silica was washed several times with hexane to remove unencapsulated phenol from the helical silica nanocavity. The silica was dispersed in a MeOH/H_2_O mixture, and electronic circular dichroism (ECD) measurements were subsequently performed. Figure 2 shows the ECD spectra of chiral silica/phenol, chiral silica, and phenol. For the chiral silica/phenol sample, a clear split-type Cotton effect was observed in the broad UV absorption peaks at 210–220 and 260–280 nm, which corresponded to the π–π* electronic transition of phenol. In contrast, the Cotton effect was not observed in this UV region for chiral silica and phenol individually (Figure 2b,c). The UV peaks in the 210–220 and 260–280 nm regions were broadened and slightly shifted to higher wavelengths (Figure 2d,e). These results indicated that the phenol molecules were placed in the helical nanocavities via *J*-aggregation, as shown in Figure 2f. Achiral chromophores can be placed along helically structured chiral silica, leading to induced ECD (iECD) in the ground state. To confirm the universality of the chirality behavior, an *S*-silica sample was immersed in 0.5 wt % pyranine/H_2_O for 4 days and subsequently rinsed twice with pure H_2_O. The induced negative Cotton effect observed at 280 nm was attributed to the characteristic absorption band along the short axis of the molecular plane of the pyrene moiety between 250 and 300 nm^18−20^ (Figure S3a). These results indicated that the *S*-silica encapsulated the pyranine molecules within its helical nanocavity (see the Supporting Information). In addition, the chiral silica exhibited no absorption peak in the UV region (Figure 2b). Thus, chiral silica has the potential to act as a nanosized fused quartz cell.

**Figure 2:**
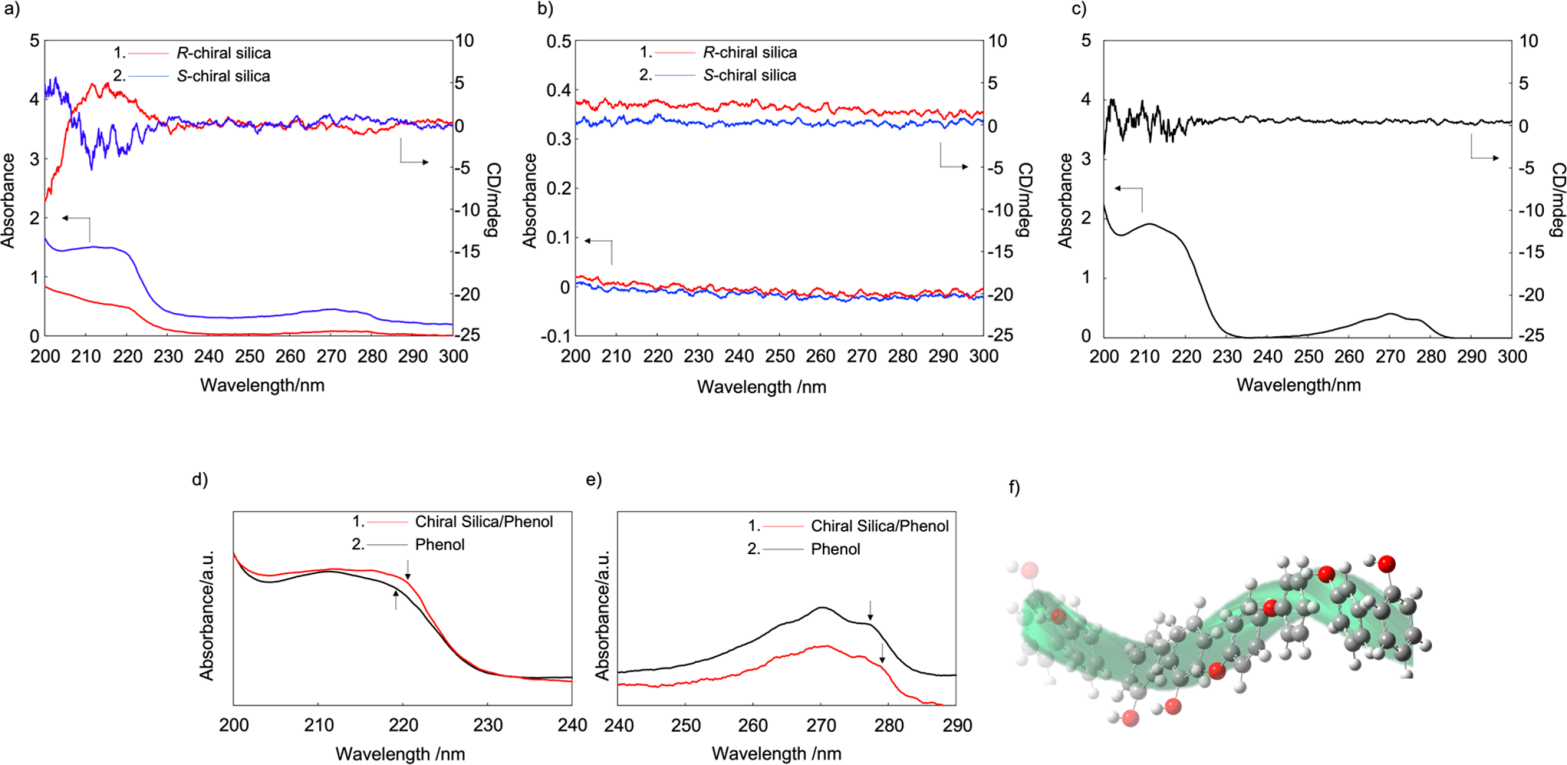
(a–c) ECD spectra of (a) chiral silica/phenol, (b) chiral silica, and (c) phenol measured in MeOH/H_2_O solution. (d, e) Highly magnified UV–vis spectra of chiral silica/phenol and phenol in MeOH/H_2_O solution. (f) Schematic of the *J*-aggregation state of phenol in a chiral silica helical nanocavity.

Because achiral chromophores can be placed in a helical nanocavity, the induced chirality of electronically excited fluorophores is also expected to occur, resulting in induced circularly polarized luminescence (iCPL). Achiral pyranine is known to exhibit specific fluorescence emissions depending on its acid dissociation state and was selected as the achiral PL molecule (Figure 3a). Pyranine was dissolved in MeOH and H_2_O to obtain 0.5 wt % clear solutions, and the chiral silica was immersed in these solutions for 2 days at room temperature. The chiral silica immersed in the pyranine/MeOH and pyranine/H_2_O solutions exhibited blue and green emissions, respectively, upon UV irradiation at 340 nm (Figure 3b). CPL measurements were performed to evaluate the effect of the solvent on the iCPL. Figure 3c shows the CPL spectra of the enantiomeric chiral silicas in the pyranine/MeOH and pyranine/H_2_O solutions. Symmetrical mirror-image CPL spectra were obtained for the enantiomeric chiral silica/pyranine in MeOH and H_2_O. This suggested that the achiral luminophores were placed along the helically structured chiral silica, leading to iCPL in the excited state. The chiral silica immersed in the pyranine/H_2_O solution exhibited a green CPL at 520 nm, whereas that in the pyranine/MeOH solution exhibited a blue CPL at 436 nm. In contrast, pyranine itself exhibited no CPL when dissolved in H_2_O or MeOH (Figure S1). The OH group in pyranine does not dissociate in MeOH, whereas dissociation occurs when pyranine is dissolved in H_2_O. The emission peaks at 436 and 520 nm were assigned to the pyranine–OH and pyranine–O^–^, respectively.^21,22^

**Figure 3:**
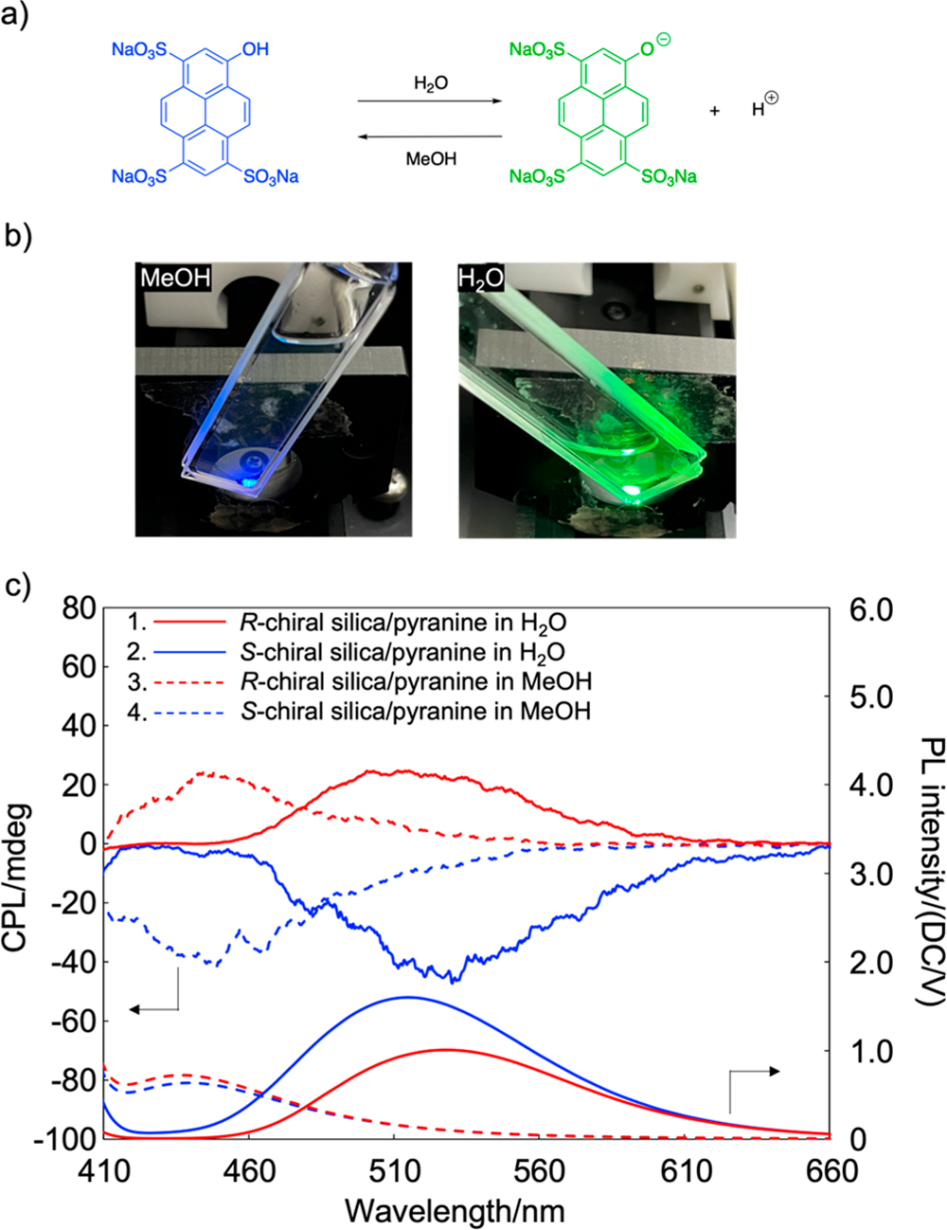
(a) Chemical structure of pyranine. (b) Photographs of chiral silica/pyranine in MeOH (left) and H_2_O (right) upon UV irradiation at 340 nm. (c) CPL and corresponding PL spectra of chiral silica associated with pyranine under excitation at 340 nm.

Considering these results and those reported in the literature, the chiral silica clearly acted as a helical nanosized fused quartz cell that encapsulated the pyranine and solvent molecules. To investigate stability of the solution within the nanosized fused quartz cell, *S*-silica with pyranine/MeOH was rinsed twice with fresh H_2_O, and the CPL dissymmetry factor (|*g*_lum_|) was evaluated (Figure 4a). The value at 436 nm increased from ∼0.0013 to ∼0.026 after two consecutive solvent replacements, which corresponded to a 20-fold enhancement. These results indicated that pyranine and MeOH molecules were accommodated within the chiral silica and that unassociated pyranine molecules could be removed by rinsing with fresh H_2_O.

**Figure 4:**
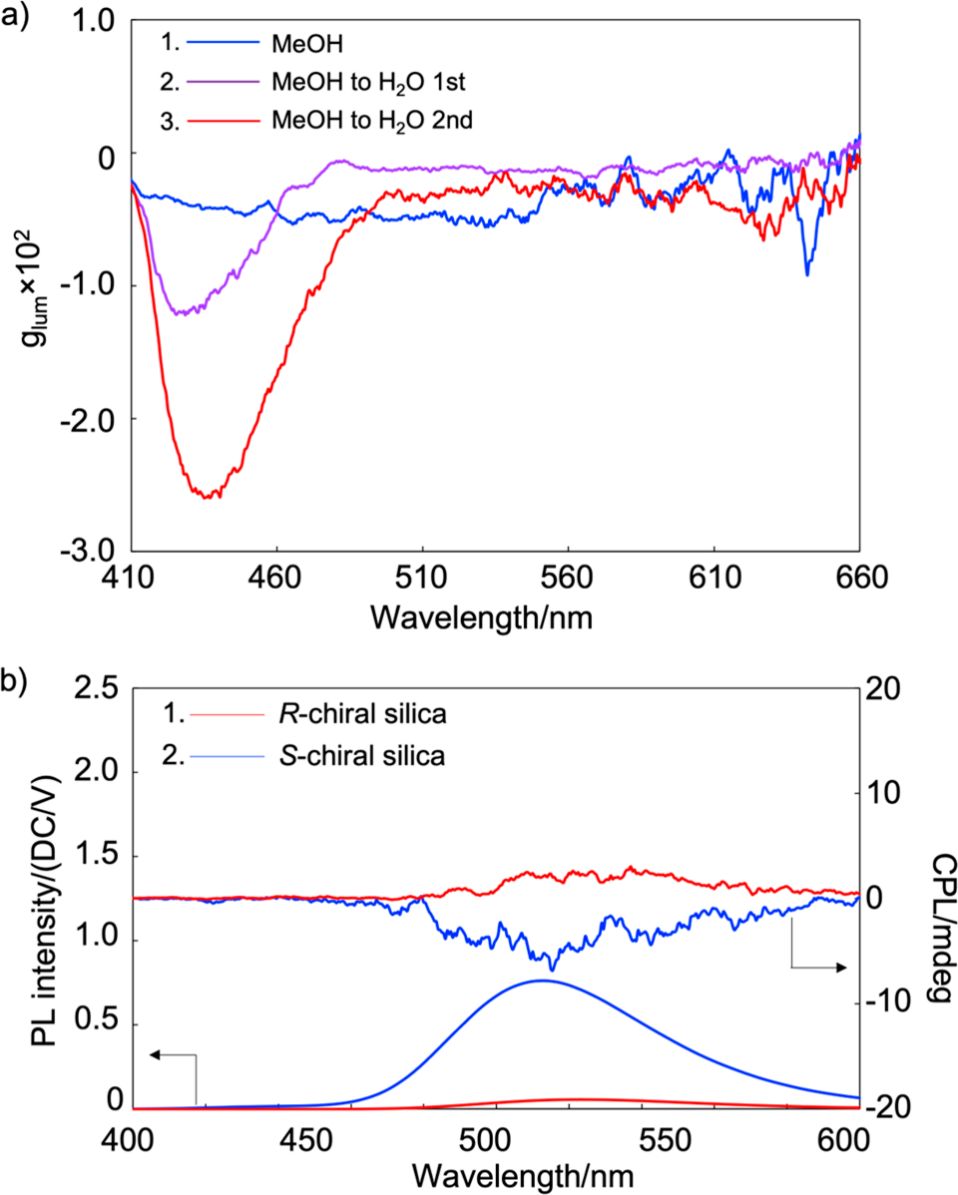
(a) *g*_lum_ spectra of *S*-silica/pyranine in MeOH and H_2_O upon UV irradiation at 340 nm. (b) CPL and corresponding PL spectra of dry-state chiral silica associated with pyranine under excitation at 340 nm.

Fluorophores typically undergo self-aggregation in the solid state, leading to ACQ (Movie S1). Therefore, the PL and CPL of chiral silica and pyranine in the solid state were evaluated. Chiral silica was immersed in a 1 wt % pyranine/H_2_O solution for 2 days at room temperature and then drop-cast onto quartz substrates; CPL measurements were subsequently performed (Figure 4b). Symmetrical mirror-image CPL spectra were observed for solid-state enantiomeric chiral silica. This indicated that the chiral silica acted as a nanosized fused quartz cell and regulated the self-aggregation of the pyranine molecules, thereby regulating the ACQ.

In conclusion, the optical activities in the electronic ground and excited states of a novel chiral silica prepared from *it*-PMAPOSS were investigated. The chiral silica acted as a nanosized fused quartz cell to encapsulate functional materials and solvents along the helical nanocavities without any surface modification. The chiral silica with functional molecules exhibited ICD and iCPL. The wavelength of the CPL between 436 and 520 nm could be controlled by changing the solvent in the achiral luminophore-containing helical nanosized fused quartz cell. This finding enables the control of the wavelength in CPL using a simple and cost-effective approach that can advance the fabrication of next-generation sensor materials for use in evaporation and separation applications.

## Supporting Information Available

Synthesis method, experimental methods, and data analysis (PDF)
Movie S1 (MP4)
Movie S2 (MP4)

## References

[ref1] Jung J. H.; Ono Y.; Hanabusa K.; Shinkai S. Creation of Both Right-Handed and Left-Handed Silica Structures by Sol-Gel Transcription of Organogel Fibers Comprised of Chiral Diaminocyclohexane Derivatives. J. Am. Chem. Soc. 2000, 122 (20), 5008–5009. 10.1021/ja000449s.

[ref2] Che S.; Liu Z.; Ohsuna T.; Sakamoto K.; Terasaki O.; Tatsumi T. Synthesis and characterization of chiral mesoporous silica. Nature 2004, 429 (6989), 281–284. 10.1038/nature02529. 15152248

[ref3] Okazaki Y.; Buffeteau T.; Siurdyban E.; Talaga D.; Ryu N.; Yagi R.; Pouget E.; Takafuji M.; Ihara H.; Oda R. Direct Observation of Siloxane Chirality on Twisted and Helical Nanometric Amorphous Silica. Nano Lett. 2016, 16 (10), 6411–6415. 10.1021/acs.nanolett.6b02858. 27585220

[ref4] Matsukizono H.; Jin R.-H. High-Temperature-Resistant Chiral Silica Generated on Chiral Crystalline Templates at Neutral pH and Ambient Conditions. Angew. Chem., Int. Ed. 2012, 51 (24), 5862–5865. 10.1002/anie.201108914. 22539201

[ref5] Tsunega S.; Jin R.-H.; Nakashima T.; Kawai T. Transfer of Chiral Information from Silica Hosts to Achiral Luminescent Guests: a Simple Approach to Accessing Circularly Polarized Luminescent Systems. ChemPlusChem 2020, 85 (4), 619–626. 10.1002/cplu.201900615. 32237231

[ref6] Cui M.; Zhang W.; Xie L.; Chen L.; Xu L. Chiral mesoporous silica materials: A review on synthetic strategies and applications. Molecules 2020, 25 (17), 3899 10.3390/molecules25173899. 32867051 PMC7504517

[ref7] Hirai T.; Leolukman M.; Liu C. C.; Han E.; Kim Y. J.; Ishida Y.; Hayakawa T.; Kakimoto M.; Nealey P. F.; Gopalan P. One-Step Direct-Patterning Template Utilizing Self-Assembly of POSS-Containing Block Copolymers. Adv. Mater. 2009, 21 (43), 4334–4338. 10.1002/adma.200900518. 26042939

[ref8] Iwao S.; Kurono N.; Higashiguchi W.; Hayakawa T.; Ohta N.; Kamitani K.; Fujii S.; Nakamura Y.; Hirai T. Ordered Silica Nanostructure by the Calcination of Block Copolymer with Polyhedral Oligomeric Silsesquioxane (POSS) Side Chain. Chem. Lett. 2022, 51 (7), 781–783. 10.1246/cl.220180.

[ref9] Guo Q.-Y.; Yan X.-Y.; Zhang W.; Li X.-H.; Xu Y.; Dai S.; Liu Y.; Zhang B.-x.; Feng X.; Yin J.; Han D.; Huang J.; Su Z.; Liu T.; Huang M.; Hsu C.-H.; Cheng S. Z. D. Ordered Mesoporous Silica Pyrolyzed from Single-Source Self-Assembled Organic-Inorganic Giant Surfactants. J. Am. Chem. Soc. 2021, 143 (33), 12935–12942. 10.1021/jacs.1c05356. 34387467

[ref10] Daga V. K.; Anderson E. R.; Gido S. P.; Watkins J. J. Hydrogen Bond Assisted Assembly of Well-Ordered Polyhedral Oligomeric Silsesquioxane-Block Copolymer Composites. Macromolecules 2011, 44 (17), 6793–6799. 10.1021/ma200926n.

[ref11] Hirai T.; Leolukman M.; Hayakawa T.; Kakimoto M.; Gopalan P. Hierarchical nanostructures of organosilicate nanosheets within self-organized block copolymer films. Macromolecules 2008, 41 (13), 4558–4560. 10.1021/ma800872v.

[ref12] Hirai T.; Leolukman M.; Jin S.; Goseki R.; Ishida Y.; Kakimoto M.; Hayakawa T.; Ree M.; Gopalan P. Hierarchical Self-Assembled Structures from POSS-Containing Block Copolymers Synthesized by Living Anionic Polymerization. Macromolecules 2009, 42 (22), 8835–8843. 10.1021/ma9018944.

[ref13] Tsai S.-Y.; Kuretani S.; Manabe K.; Terao T.; Komamura T.; Agata Y.; Ohta N.; Fujii S.; Nakamura Y.; Wang C.-L.; Hayakawa T.; Hirai T. Preparation of polyhedral oligomeric silsesquioxane-containing block copolymer with well-controlled stereoregularity. J. Polym. Sci., Part A: Polym. Chem. 2019, 57 (21), 2181–2189. 10.1002/pola.29498.

[ref14] Kometani S.; Kato T.; Manabe K.; Seko T.; Chang Y.-N.; Luo H.-R.; Agata Y.; Ohta N.; Hayakawa T.; Fujii S.; Nakamura Y.; Li M.-C.; Hirai T. Preferred-handed helical conformation in organic-inorganic hybrid block copolymers with well-controlled stereoregularity. J. Polym. Sci. 2022, 60 (5), 766–773. 10.1002/pol.20210761.

[ref15] Huang M.; Hsu C.-H.; Wang J.; Mei S.; Dong X.; Li Y.; Li M.; Liu H.; Zhang W.; Aida T.; Zhang W.-B.; Yue K.; Cheng S. Z. D. Selective assemblies of giant tetrahedra via precisely controlled positional interactions. Science 2015, 348 (6233), 424–428. 10.1126/science.aaa2421. 25908818

[ref16] Zong Z.; Hao A.; Xing P.; Zhao Y. Chiral molecular nanosilicas. Chem. Sci. 2022, 13 (14), 4029–4040. 10.1039/D2SC00793B. 35440995 PMC8985511

[ref17] Manabe K.; Tsai S.-Y.; Kuretani S.; Kometani S.; Ando K.; Agata Y.; Ohta N.; Chiang Y.-W.; Lin I. M.; Fujii S.; Nakamura Y.; Chang Y.-N.; Nabae Y.; Hayakawa T.; Wang C.-L.; Li M.-C.; Hirai T. Chiral Silica with Preferred-Handed Helical Structure via Chiral Transfer. JACS Au 2021, 1 (4), 375–379. 10.1021/jacsau.1c00098. 34467302 PMC8395658

[ref18] Thomas J. K.; Richards J. T.; West G. Formation of ions and excited states in the laser photolysis of solutions of pyrene. J. Phys. Chem. 1970, 74 (23), 4137–4141. 10.1021/j100717a023.

[ref19] Porter G.; Topp M. R. Nanosecond flash photolysis. Proc. R. Soc. London, Ser. A 1970, 315 (1521), 163–184. 10.1098/rspa.1970.0035.

[ref20] Kalyanasundaram K.; Thomas J. K. Environmental effects on vibronic band intensities in pyrene monomer fluorescence and their application in studies of micellar systems. J. Am. Chem. Soc. 1977, 99 (7), 2039–2044. 10.1021/ja00449a004.

[ref21] Kondo H.; Miwa I.; Sunamoto J. Biphasic structure model for reversed micelles. Depressed acid dissociation of excited-state pyranine in the restricted reaction field. J. Phys. Chem. 1982, 86 (24), 4826–4831. 10.1021/j100221a035.

[ref22] Pouxviel J. C.; Dunn B.; Zink J. I. Fluorescence study of aluminosilicate sols and gels doped with hydroxy trisulfonated pyrene. J. Phys. Chem. 1989, 93 (5), 2134–2139. 10.1021/j100342a082.

