# Supplementary material for: Controlling Circularly Polarized Luminescence Using Helically Structured Chiral Silica as a Nanosized Fused Quartz Cell
Source: JACS Au. 2023 Sep 15;3(10):2698–702. doi: 10.1021/jacsau.3c00390 (PMC10598828; doi:10.1021/jacsau.3c00390)
Supplement: Supplementary file 1 — au3c00390_si_001.pdf [file au3c00390_si_001.pdf]

Supporting Information

Controlling Circularly Polarized Luminescence using Helically Structured Chiral Silica as a Nano-Sized Fused Quartz Cell

Hinari Sakai[†], Tsz-Ming Yung[§], Tomoki Mure[†], Naoki Kurono[†] Syuji Fujii[†], Yoshinobu Nakamura[†], Teruaki Hayakawa[‡], Ming-Chia Li[§]*, and Tomoyasu Hirai[†]*

[†]Department of Applied Chemistry, Faculty of Engineering and Graduate School of Engineering

Osaka Institute of Technology, 5-16-1 Omiya, Asahi-ku, Osaka 535-8585, Japan

[§]Department of Biological Science and Technology, Center For Intelligent Drug Systems and Smart Bio-devices (IDS2B)

National Yang Ming Chiao Tung University, Hsinchu 30010, Taiwan

[‡]Department of Materials Science and Engineering, School of Materials and Chemical Technology

Tokyo Institute of Technology, 2-12-1-S8-36 Ookayama, Meguro-ku, Tokyo 152-8552, Japan

Correspondence to: T. Hirai (E-mail: tomoyasu.hirai@oit.ac.jp), M. -C. Li (E-mail: mingchiali@nycu.edu.tw)

Contents.

1. Materials and Methods p4–8

1-1. Materials

1-2. Instrumentation

1-3. Preparation of *it*-PMAPOSS

1-4. Calcination of *it*-PMAPOSS with Enantiomeric BN

1-5. Accommodation of Phenol in Chiral Silica with Helical Nanocavity

1-6. CPL and ECD Measurements of Pyranine in Chiral Silica containing Solvent Molecules

1-7. CPL Measurements of Pyranine in Chiral Silica in Solid State

2. Supporting Figures p9–14

Figure S1. a) CPL and b) PL of pyranine in MeOH and H_2O. c) Photographs of 0.5 wt% pyranine solutions in MeOH and H_2O upon UV irradiation at 340 nm.

Figure S2. a) Photograph of *S*-chiral silica/pyranine in MeOH/H_2O (50/50 v/v). b) CPL and corresponding PL spectra of s-chiral silica associated with pyranine under excitation at 340 nm.

Figure S3. a) ECD spectra of chiral silica immersed in pyranine/H_2O solution (black line) and subsequently rinsed twice using pure H_2O (blue and red lines). b) ECD spectra of chiral silica immersed in pyranine/MeOH solution (black line) and subsequently rinsed twice using pure MeOH (blue and red lines).

Figure S4. DRCD and corresponding visible absorption spectra of chiral silica prepared using pyranine/H_2O solution (blue line) and pyranine/MeOH solution (red lines). The black line denotes blank quartz.

Figure S5. Time-dependent photoluminescent spectra of 0.5 wt% pyranine/MeOH solution dropped onto *S*-chiral silica powder under excitation at 340 nm.

Figure S6. g_{lum} spectra of *R*- and *S*-chiral silica in solid state.

Movie S1. 0.5 wt% pyranine/MeOH solution dropped onto *S*-chiral silica powder and glass slide during MeOH evaporation.

Movie S2. ACQ effect of pyranine film casted using H_2O, H_2O/MeOH (50/50 v/v), and MeOH.

3. Reference p15

1. Materials and Methods

1-1. Materials

All chemicals were used without further purification except for 3-(3,5,7,9,11,13,15-heptaisobutylpentacyclo [9.5.1^{3,9}.1^{5,15}1^{7,13}] octasiloxan-1-yl) propylmethacrylate (MAPOSS; Hybrid Plastics) and toluene (98.0%; FUJIFILM Wako Pure Chemical Corporation,). Magnesium turnings (99.5%), calcium hydride (CaH_2, 95.0%), and diethyl ether (99.0%) were purchased from FUJIFILM Wako Pure Chemical Corporation. *n*-Butyllithium (*n*-BuLi, 1.5 M in cyclohexane) was purchased from Kanto Chemical Industry Co., Ltd. 1,1-Diphenyl ethylene (DPE, 98.0%), 2-bromo-2-methylpropane (98.0%), (*R*)-(+)-5,5',6,6',7,7',8,8'-octahydro-1,1'-bi-2-naphthol ((*R*)-BN, 99.0%), and (*S*)-(-)-5,5',6,6',7,7',8,8'-octahydro-1,1'-bi-2-naphthol ((*S*)-BN, 99.0%) were purchased from Tokyo Chemical Industry Co., Ltd.

1-2. Instrumentation

^{1}H (400MHz) and ^{13}C NMR (100 MHz) spectra were recorded in $CDCl_3$ using a JEOL JNM-ECZ400S spectrometer. The number average molecular weight (M_n) and polydispersity index ($Đ$) were determined by size exclusion chromatography (SEC) using a 1260 Infinity II instrument (Agilent) with three columns (TSKgel Super H2500, TSKgel Super H4000, and TSKgel Super H6000; Tosoh) and a 1260 refractive index (RI) detector. THF was used as the eluent at a flow rate of 0.5 mL min^{-1}, and the measurements were performed at 40°C. Circular dichroism (CD), vibrational circular dichroism (VCD), and circularly polarized luminescence (CPL) measurements were performed using J-720W (CD; JASCO), J-1700 (CD; JASCO), VFT-4000 (VCD; JASCO), and CPL-300 (JASCO) spectrometers, respectively.

1-3. Preparation of *it*-PMAPOSS

it-PMAPOSS was prepared according to the method described in a previous report.[1] Briefly, 54 mL of diethyl ether and 3.3 g (135 mmol) of magnesium turnings were placed in a three-neck flask with a dropping funnel. Then, 12.3 g (90.0 mmol) of 2-bromo-2-methylpropane was diluted with 27 mL of diethyl ether and placed in the dropping funnel. The mixture was slowly added to the three-neck flask under an Ar atmosphere, and the

resulting solution was stirred for 12 h. The obtained Grignard reagent was stored in a Schlenk flask at 0°C until use.

In a Schlenk flask, 10 mL of toluene and 0.5 mL of Grignard reagent were mixed and cooled to −78°C. Then, 1.00 g (1.06 mmol) of MAPOSS was dissolved in 3 mL of toluene and the solution was added to the Schlenk flask under vigorous stirring. The polymerization reaction was maintained at −78°C for 120 h, and 1 mL of MeOH was subsequently added to the flask. The mixture was precipitated in MeOH. Finally, 0.72 g of *it*-PMAPOSS was obtained as a white powder. The M_n and PDI were 8,000 and 1.24, respectively. ^{1}H NMR (400 MHz, $CDCl_3$, δ, ppm): 3.74 (s, –OCH_2–), 2,11 (br, –CH_2–, main chain), 1.97–1.75 (br, –CH_2, main chain, CH, isobutyl in PMAPOSS), 1.65 (br, –CH_2–, in ester side chain), 1.16 (br, α-CH_3), 1.05–0.86 (be, isobutyl CH_3 in PMAPOSS), 0.68–0.50 (br, Si–CH_2–). ^{13}C NMR (75 MHz, $CDCl_3$, δ, ppm): 175.5, 66.7, 52.5, 45.7, 25.7, 23.8, 21.7, 8.9.

1-4. Calcination of *it*-PMAPOSS with enantiomeric BN

Chiral silica was prepared using the method described in the previous report.[1] *it*-PMAPOSS and BN were mixed at a molar ratio of MAPOSS/BN = 0.1 in toluene, and the solution was annealed at 90° for 2 h. The mixture was then stored at room temperature for 48 h before the toluene was removed using a rotary evaporator. The sample thus obtained was calcinated at 620 °C under atmospheric conditions. The optical properties of the chiral silica were evaluated using VCD measurements. As shown in the main text, a symmetrical mirror-imaged split-type Cotton effect was observed in the enantiomeric chiral silica spectra. Taking this and our previous results into account, we concluded that the chiral silica formed a preferred-handed helical conformation.

1-5. Accommodation of Phenol in the Chiral Silica with Helical Nanocavity

For this experiment, 5 mg of enantiomeric chiral silica was immersed in 1 mL of a 5 mM phenol/hexane solution for 14 h. The chiral silica was then washed using pure hexane to remove excess phenol.

1-6. CPL and ECD Measurements of Pyranine in Chiral Silica containing Solvent Molecules

The effect of the solvent on the CPL and PL emissions of pyranine was evaluated by

immersing 5 mg of enantiomeric chiral silica in 1 mL of 0.5 wt% solutions of pyranine in H_2O and MeOH for 2 d. The CPL and PL were evaluated at an excitation wavelength of 340 nm. As shown in the main text, symmetrical mirror-imaged CPL were observed, indicating that the pyranine and solvent molecules were placed within the helically structured chiral silica. Figure S1 shows the CPL and PL of 0.5 wt% pyranine in H_2O and MeOH solutions. The pyranine/H_2O solution exhibited a PL at 520 nm, while the pyranine/MeOH solution exhibited a PL at 436 nm. No CPL was observed in pyranine solution (Figure S1). CPL control of pyranine in chiral silica using mixture solvent was also performed. During the process, 5 mg of *S*-chiral silica was immersed in 1 mL of 0.5 wt% solutions of pyranine H_2O/MeOH (50/50, v/v) mixture solution for 2 d. The CPL and PL were evaluated at an excitation wavelength of 340 nm. The chiral silica exhibited a distinct light-green CPL peak at 506 nm (Figure S2).

To confirm the encapsulation behavior, the *S*-silica sample was immersed in 0.5 wt% pyranine/H_2O for 4 d and subsequently rinsed twice with pure H_2O. The black line in Figure S3a shows the ECD spectrum of *S*-silica after immersion in pyranine/H_2O for 4 d. A negative Cotton effect was observed for the absorption peak at 280 nm. This result indicated that the *S*-silica encapsulated the pyranine molecules in its helical nanocavity. The blue and red lines in Figure S3a show the ECD spectra after the first and second treatments, respectively, with pure H_2O. The CD signals in the ECD spectra of the untreated and treated samples were comparable, while the UV intensity decreased. This indicated that the *g*-factor of the treated sample was significantly higher than that of the untreated sample, suggesting that pyranine that was not encapsulated within the helical nanocavity was removed during this treatment. The encapsulation behavior of *S*-silica in MeOH was confirmed by immersing an *S*-silica sample in 0.5 wt% pyranine/MeOH for 4 d and subsequently rinsing the sample twice with pure MeOH. The black line in Figure S3b shows the ECD spectrum of *S*-silica after immersion in pyranine/MeOH for 4 d. A negative Cotton effect was observed for the absorption peak at 280 nm. This result indicated that the *S*-silica encapsulated the pyranine molecules in its helical nanocavity. The blue and red lines in Figure S3b show the ECD spectra after the first and second treatments, respectively, using pure MeOH. Both the CD and UV intensity decreased during this treatment, indicating that MeOH removed the encapsulated pyranine from the chiral silica. Hence, H_2O was selected as the washing solvent. Diffuse reflectance CD

(DRCD) measurement was also performed to evaluate ECD on visible region (Figure S4). Negative cotton effect, as seen in Figure S3, was observed at 280 nm. Although the absorption and CD signal were weak, DRCD signal was observed in range 450–650 nm. The characterization absorption of pyranine was very weak attributed to the limited optical penetration depth of the DRCD method and the DRCD signal arising from the diffuse reflectance (i.e. interface reflection attributed to the reflective index change).

1-7. CPL Measurements of Pyranine in Chiral Silica at Solid State.

In general, ACQ occurs when high concentration of luminophore is present. A 5 mg of *S*-chiral silica was immersed in 1 mL of a 0.5 wt% pyranine/MeOH solution to emphasize the regulating ACQ effect. The sample was then casted onto a quartz substrate. The pyranine molecules did not exhibit PL and CPL in the solid state because of ACQ (Movie S1). Although pyranine film prepared on quartz substrate did not show any PL, clear PL is observed in the pyranine accommodated in chiral silica (Movie S1). These results indicated that ACQ was regulated in the chiral silica. ACQ occurs rapidly in the case pyranine casted using MeOH. In contrast, owing to the slower evaporation rate of H_2O, ACQ effect extends over longer period (Movie S2, left: H_2O, center: H_2O/MeOH (50/50 v/v), right: MeOH). Here, we should note that ACQ also occurs in pyranine casted from H_2O. (The ACQ takes place in 1 h.) Therefore, MeOH was used to demonstrate dynamic ACQ regulation behavior of the chiral silica using the movie, as shown in Movie S1.

Another experiment was performed to understand why chiral silica with pyranine prepared using MeOH solution showed green emission. 0.5 wt% pyranine/MeOH solution is casted on a *S*-chiral silica and subsequently time-dependent photoluminescence measurements is performed (Figure S5). Blue emission peak at 436 nm gradually decreased and green emission at 520 nm increased with time. When MeOH evaporate, the MeOH solvent molecules absorbs heat from surrounding consequently decreasing the surface temperature of the MeOH solution. Meanwhile, the water vapor condenses and dissolves in the MeOH solution. Consequently, the solution becomes H_2O/MeOH mixing solvent during MeOH evaporation. Therefore, the emission color in dry process changed from blue to green.

The ACQ behavior of chiral silica with pyranine prepared using H_2O was also

investigated. 5 mg of enantiomeric chiral silica was immersed in 1 mL of a 1 wt% pyranine/H_2O solution. The sample was then casted onto a quartz substrate. The chiral silica was washed with pure H_2O to remove excess pyranine. The sample was set at room temperature for at least 8 h before CPL measurements. The CPL and PL were evaluated at an excitation wavelength of 340 nm. Symmetrical mirror-imaged CPL was observed at 520 nm (**Figure 4 in main text**). These indicates that ACQ was regulated using chiral silica, which acts as a nano-sized fused quartz cell. Figure S6 shows the g_{lum} spectrum of solid-state *R* and *S* chiral silica immersed in H_2O/pyranine. The g_{lum} at 520 nm is ~ 0.0004 to ~ 0.003, smaller in magnitude than that in solution state. This finding suggests that solvent molecules in chiral silica strongly affects g_{lum}.

2. Supporting Figures

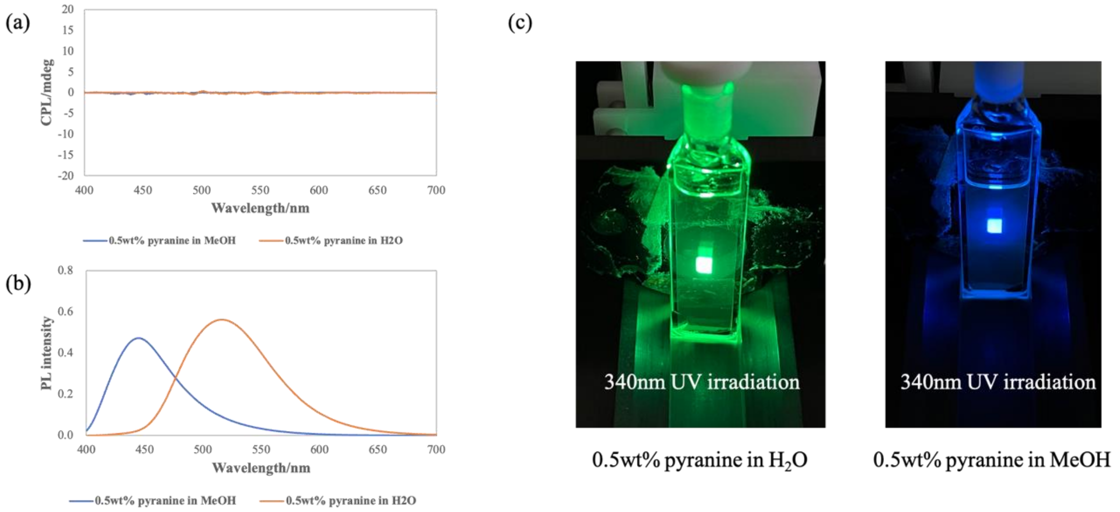

Figure S1. a) CPL and b) PL of pyranine in MeOH and H_2O. c) Photographs of 0.5 wt% pyranine solutions in MeOH and H_2O upon UV irradiation at 340 nm.

a)

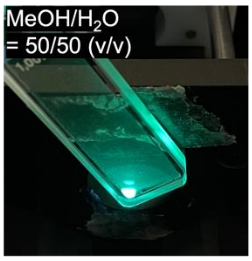

b)

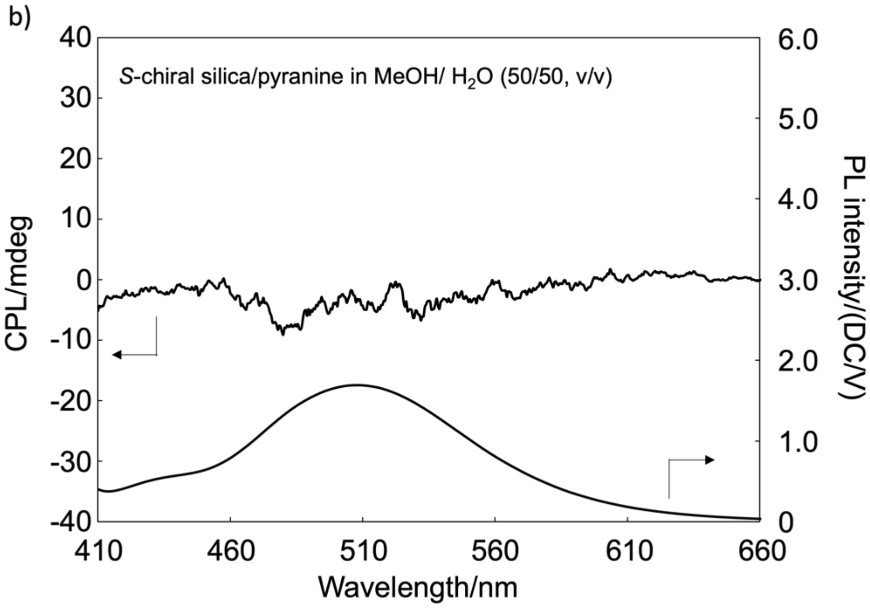

Figure S2. a) Photograph of *S*-chiral silica/pyranine in MeOH/H_2O (50/50 v/v). b) CPL and corresponding PL spectra of s-chiral silica associated with pyranine under excitation at 340 nm.

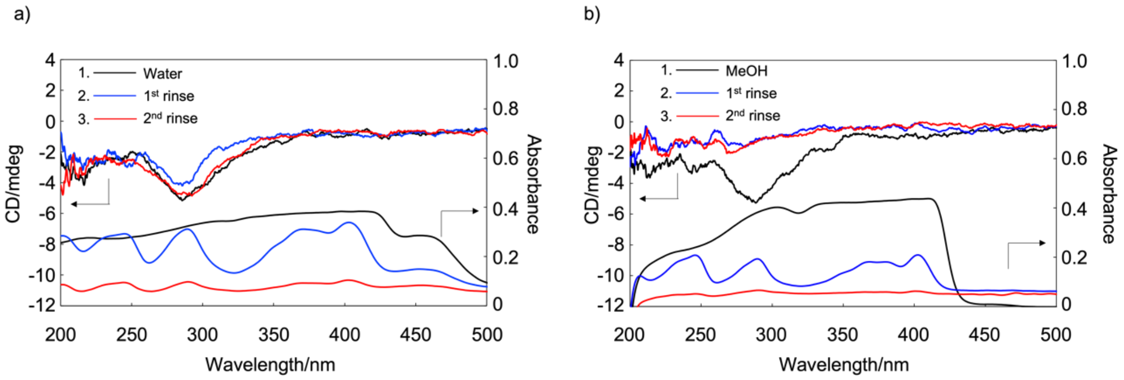

Figure S3. a) ECD spectra of chiral silica immersed in pyranine/H_2O solution (black line) and subsequently rinsed twice using pure H_2O (blue and red lines). b) ECD spectra of chiral silica immersed in pyranine/MeOH solution (black line) and subsequently rinsed twice using pure MeOH (blue and red lines).

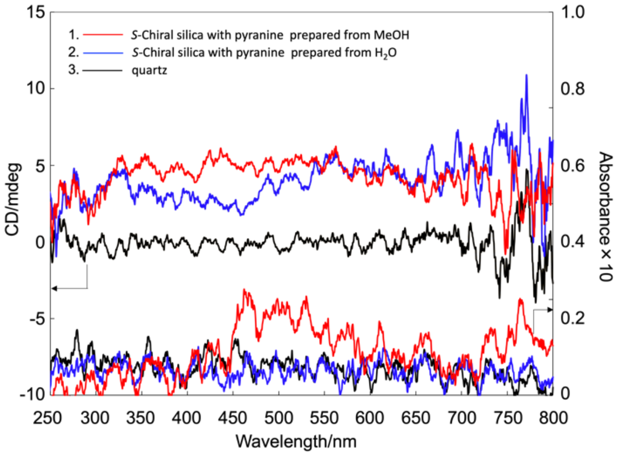

Figure S4. DRCD and corresponding visible absorption spectra of chiral silica prepared using pyranine/H_2O solution (blue line) and pyranine/MeOH solution (red lines). The black line denotes blank quartz.

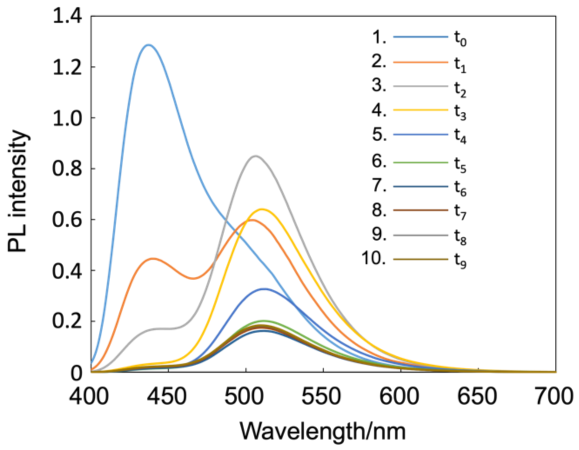

Figure S5. Time-dependent photoluminescent spectra of 0.5 wt% pyranine/MeOH solution dropped onto *S*-chiral silica powder under excitation at 340 nm.

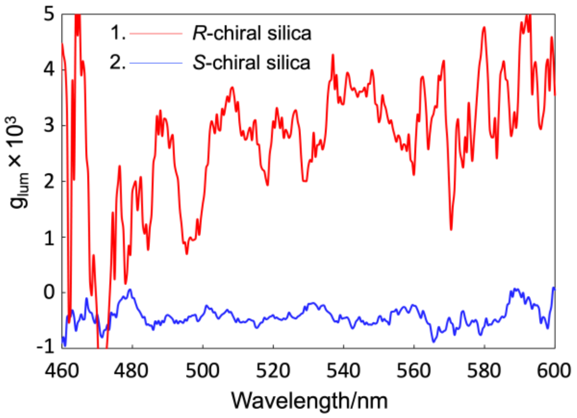

Figure S6. g_{lum} spectra of *R*- and *S*-chiral silica in solid state.

3. Reference

[1] Manabe, K.; Tsai, S.-Y.; Kuretani, S.; Kometani, S.; Ando, K.; Agata, Y.; Ohta, N.; Chiang, Y.-W.; Lin, I. M.; Fujii, S.; Nakamura, Y.; Chang, Y.-N.; Nabae, Y.; Hayakawa, T.; Wang, C.-L.; Li, M.-C.; Hirai, T., Chiral Silica with Preferred-Handed Helical Structure via Chiral Transfer. *JACS Au* **2021**, *1*(4), 375–379.
